# Antibiotic consumption in French nursing homes between 2018 and 2022: A multicenter survey

**DOI:** 10.1017/ice.2024.19

**Published:** 2024-06

**Authors:** Ségolène Bouges, Amélie Jouzeau, Florence Lieutier-Colas, Muriel Péfau, Lory Dugravot, Anne-Marie Rogues, Loic Simon, Catherine Dumartin

**Affiliations:** 1 CPIAS Nouvelle-Aquitaine, CHU de Bordeaux, Bordeaux, France; 2 CPIAS Grand-Est, CHU de Nancy, France; 3 Service d’hygiène hospitalière, CHU de Bordeaux, Bordeaux, France; 4 Univ. Bordeaux, INSERM, BPH, U1219, Team AHeaD, Bordeaux, France

## Abstract

**Objectives:**

Monitoring antibiotic consumption is a key component to steer antimicrobial stewardship programs, including in nursing homes. We analyzed changes in antibiotic consumption in French nursing homes during 5 years, including the COVID-19 pandemic, to identify potential priorities for improvement.

**Design:**

A multicenter survey was conducted between 2018 and 2022.

**Setting:**

The study was conducted across 220 French nursing homes with on-site pharmacies.

**Method:**

Antibiotic consumption data were collected from pharmacy records and are expressed as defined daily doses per 1,000 resident days. Antibiotic indicators promoted by health authorities were calculated from quantitative data to evaluate the quality of prescribing.

**Results:**

Antibiotic consumption significantly decreased between 2018 and 2022, particularly during the coronavirus disease 2019 (COVID-19) pandemic, despite a slight increase in 2022. During the study period, the most used antibiotic classes were penicillins (61.9% in 2022) followed by cephalosporins (10.5%), macrolides–lincosamides–streptogramins (7.3%) then fluoroquinolones (7.0%). Amoxicillin–clavulanic acid was the most consumed antibiotic; amoxicillin and ceftriaxone ranked second and third. Azithromycin consumption increased from 2020, as did the indicator regarding broad-spectrum antibiotics.

**Conclusions:**

The decreasing trend in antibiotic use and control of fluoroquinolone use over the study period suggest compliance with antibiotic use guidelines. However, changes in the use of broad-spectrum antibiotics and the substantial use of amoxicillin-clavulanic acid, although it is rarely a first-line antibiotic, highlight the need for antimicrobial stewardship activities and the usefulness of antibiotic consumption surveillance to identify priorities.

Antimicrobial resistance (AMR) is an increasing threat for human health around the world. In 2019, an estimated 4·95 million deaths were associated with bacterial AMR, including 1.27 million deaths attributable to bacterial AMR.^1^

Nursing home residents have a higher risk to develop infections due to bacteria resistant to antibiotics. In 2021, in France, 9.2% of *Escherichia coli* strains isolated from diagnostic samples where resistant to third-generation cephalosporins; the proportion of fluoroquinolone-resistant strains had increased since 2017 to reach 19.5%.^2^ Antibiotic use is one of the main drivers of AMR, especially misuse and overuse of broad-spectrum antibiotics.^3^ In the United States, over 1 year, up to 70% of residents receive 1 or more courses of systemic antibiotics, of which 40%–75% may be unnecessary or inappropriate.^4^ In 2016, a point-prevalence survey in French nursing homes highlighted that 2.76% of residents received an antibiotic the day of the survey and that third-generation cephalosporins was the most prescribed antibiotic class (20.9% of total prescriptions).^5^

If most countries address nursing homes in their national action plans,^6^ implementation of antimicrobial stewardship (AMS) activities remains challenging.^7^ A key component of AMS programs consists in measuring antibiotic use to identify and track quality-improvement targets.^8^ Indeed, surveillance of antibiotic use may provide helpful information to adapt AMS programs and improve practices.^9^

In this context, since 2018, the national network for surveillance and prevention of antimicrobial resistance in healthcare facilities (SPARES) has conducted a yearly survey regarding antibiotic consumption in nursing homes with on-site pharmacies. Based on the data collected between 2018 and 2022, we analyzed changes in antibiotic consumption in these facilities, including during the COVID-19 pandemic.

## Materials and methods

We conducted a retrospective survey about antibiotic consumption between 2018 and 2022 in a group of 220 nursing homes with an on-site pharmacy. French nursing homes are facilities dedicated to the care of elderly people (mean age, 85 years) who require care and daily help with everyday activities but are medically stable and do not need constant medical care. Medicines are provided by community pharmacies in 80% of nursing homes and by an on-site pharmacy in 20%. Among the 220 nursing homes included in our survey, all but 4 nursing homes were hospital based. They included only long-term care beds, and they accounted for 12,891,004 resident days in 2022.

Data collection was carried out on a voluntary basis, using a web tool according to a national methodology.^10^ Data were collected each year, retrospectively, for the whole previous year, and included information on nursing homes activity such as the number of resident days and consumption data for systemic use antibiotics, rifampicin, fidaxomicin, and oral imidazole derivatives. For each antibiotic, the number of vials and tablets dispensed by the on-site pharmacy was first converted to a number of defined daily doses (DDD), according to the World Health Organization (WHO) recommendations. The DDD is the assumed average maintenance dose per day for a medicine used for its main indication in adults.^11^ These data were standardized to the exposed population by expressing consumption as DDDs per 1,000 residents per day (DDD/1,000 RD). Total antibiotic consumption (ie, pooled mean) was calculated each year for all nursing homes, as was the consumption of specific antibiotics or antibiotic groups.

In addition, to better approach the quality of antibiotic use, we calculated indicators. First, we calculated the proportion of “critical antibiotics with restricted indications” according to a national classification provided by the French society of infectious diseases (SPILF) because of their potential to induce resistance in bacteria due to their broad-spectrum activity.^12^ These “critical antibiotics with restricted indications” include the following: amoxicillin-clavulanic acid, cefadroxil, cefalexin, cefaclor, cefuroxime, cefpodoxime, ceftriaxone, ciprofloxacin, levofloxacin, lomefloxacin, moxifloxacin, norfloxacin, ofloxacin, azithromycin, fusidic acid, thiamphenicol, rifampicin. Next, we calculated 2 other indicators proposed for nursing homes by an expert consensus group: (1) the proportion of oral cephalosporins among all cephalosporins and (2) the proportion of injectable antibiotics among systemic use antibiotics.^13^ Indeed, the use of parenteral antibiotics may expose residents to intravascular device infections, and these antibiotics are generally broad-spectrum antibiotics. Oral cephalosporins are not first-line antibiotics; cephalosporins are generally indicated for serious infections where the parenteral route is required. Trends in consumption over years were assessed using the Friedman nonparametric test for nonindependent series. Analysis were conducted using S-Plus software (Tibco, Palo Alto, CA).

## Results

### Changes in antibiotic use between 2018 and 2022

**Figure 1.**
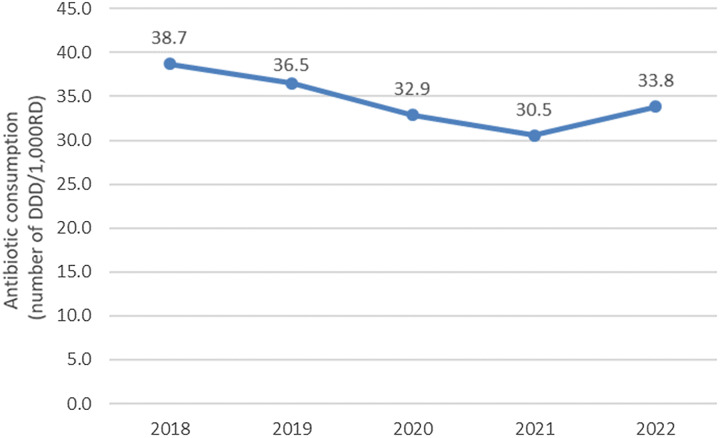
Antibiotic consumption in 220 nursing homes between 2018 and 2022. DDD/1,000 RD is the defined daily dose per 1,000 resident days.

Between 2018 and 2021 the total antibiotic consumption decreased in the 220 nursing homes, particularly during the COVID-19 pandemic (2020–2021). This steep decrease was followed by an increase in 2022 to 33.8 DDD/1,000 RD but remained below pre–COVID-19 values for 2018 and 2019. Nevertheless, the global trend showed a significant decrease in antibiotic use (*P* < .01) (Fig. 1).

In 2022, the most used antibiotic classes were penicillins (20.9 DDD/1,000 RD; 61.9%), cephalosporins (3.5 DDD/1,000 RD; 10.5%), macrolides–lincosamides–streptogramins (MLS, 2.5 DDD/1,000 RD; 7.3%), and fluoroquinolones (2.4 DDD/1,000 RD; 7.0%). This ranking was similar during the 4 previous years. The 4 most used antibiotics each year between 2018 and 2022 were amoxicillin-clavulanic acid, amoxicillin, ceftriaxone, and pristinamycin (Table 1).

**Table 1.** Consumption of the 11 Most Used Antibiotic in 220 French Nursing Homes, 2018–2022

WHO ATC Class	Antibiotic	Consumption, DDD/1,000 RD					Change 2018–2022	*P* Value^a^
		2018	2019	2020	2021	2022	(%)	
J01CR02	Amoxicillin and clavulanic acid	12.7	12.8	11.4	9.9	11.9	−6.4	<.01
J01CA04	Amoxicillin	11.1	10.1	7.6	7.2	8.0	−27.6	<.01
J01DD04	Ceftriaxone	2.5	2.2	2.3	2.0	2.2	−13.1	<.01
J01FG	Pristinamycin	1.7	1.5	1.4	1.2	1.2	−29.4	<.01
J01DD08	Cefixime	1.3	1.0	1.0	0.9	1.0	−26.0	<.01
J01MA01	Ofloxacin	1.2	1.0	1.0	1.0	0.9	−26.0	<.01
J01EE01	Sulfamethoxazole and trimethoprim	1.0	1.1	1.1	1.2	1.2	18.9	.57
J01AA02	Doxycycline	1.0	1.0	1.1	1.1	1.2	17.4	.72
J01XE01	Nitrofurantoin	0.7	0.7	0.5	0.7	0.7	0.6	…
J01MA02	Ciprofloxacin	0.7	0.6	0.7	0.7	0.7	5.3	…
J01MA12	Levofloxacin	0.5	0.6	0.7	0.8	0.7	29.9	<.01

a The *P* trend was calculated between 2018 and 2022 using Friedman test.

Among penicillins, amoxicillin and amoxicillin-clavulanic acid use followed the total consumption trend, with a decrease until 2021. Despite a rise in 2022, the global trend for these 2 antibiotics showed a significant decrease between 2018 and 2022 (*P* < .01). Among cephalosporins, ceftriaxone consumption significantly decreased between 2018 and 2022 (*P* < .01) with an increase in 2020 (2.3 DDD/1,000 RD).

Among MLS, pristinamycin consumption significantly decreased. Doxycycline consumption tended to increase, but this trend was not statistically significant. Azithromycin consumption sharply increased (+70%) between 2019 and 2020. This upward trend continued until 2022, resulting in a significant increase between 2018 and 2022.

Among fluoroquinolones, in which consumption had not significantly changed over the years, levofloxacin consumption has significantly increased, whereas the opposite is true for ofloxacin. The use of ciprofloxacin did not change over the study period. No significant change occurred in the use of sulfamethoxazole and trimethoprim nor nitrofurantoin between 2018 and 2022.

### Changes in antibiotic indicators between 2018 and 2022

**Figure 2.**
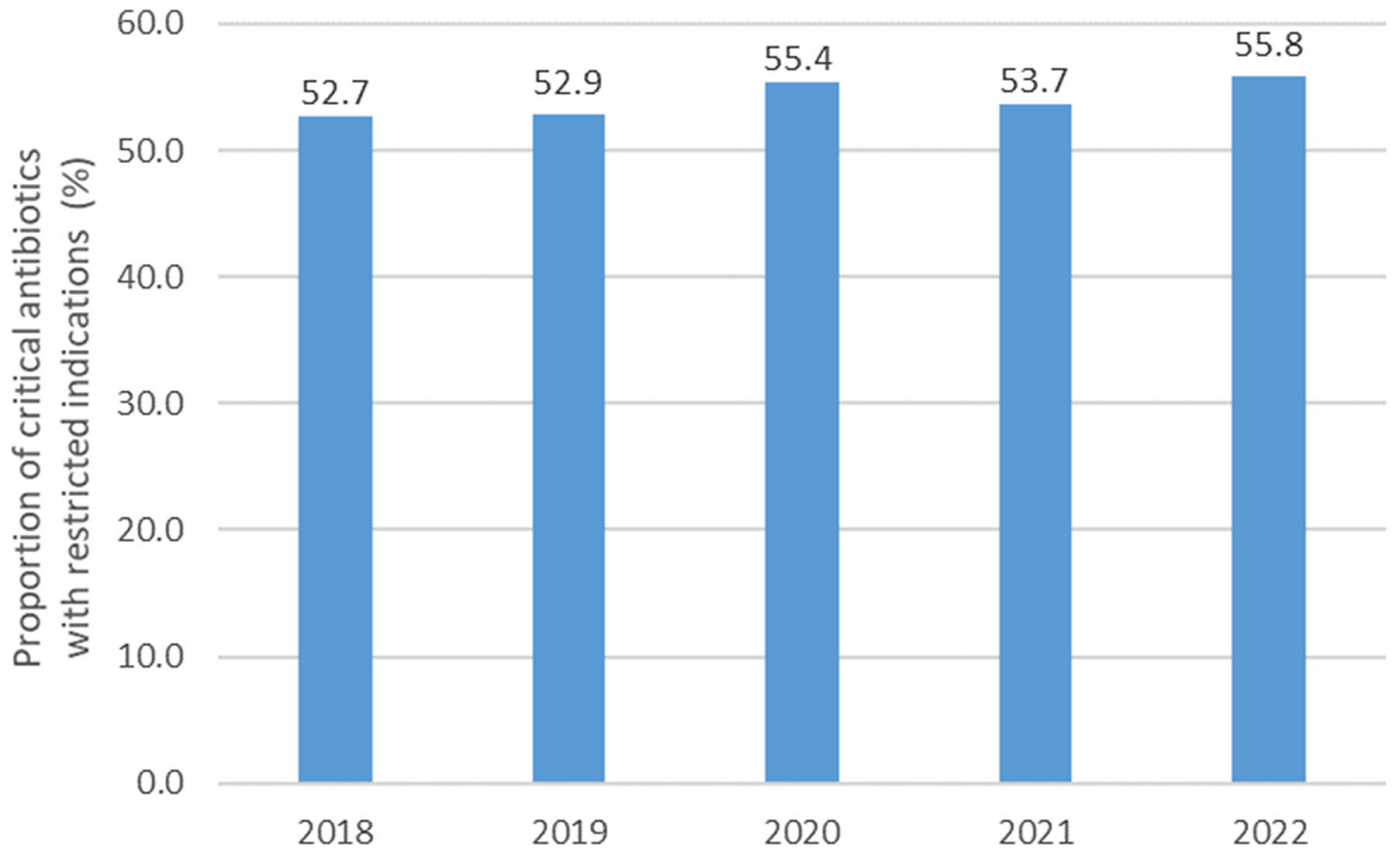
Proportion of antibiotics included in the French list of critical antibiotics with restricted indication 2018–2022 (N = 220 nursing homes).

**Figure 3.**
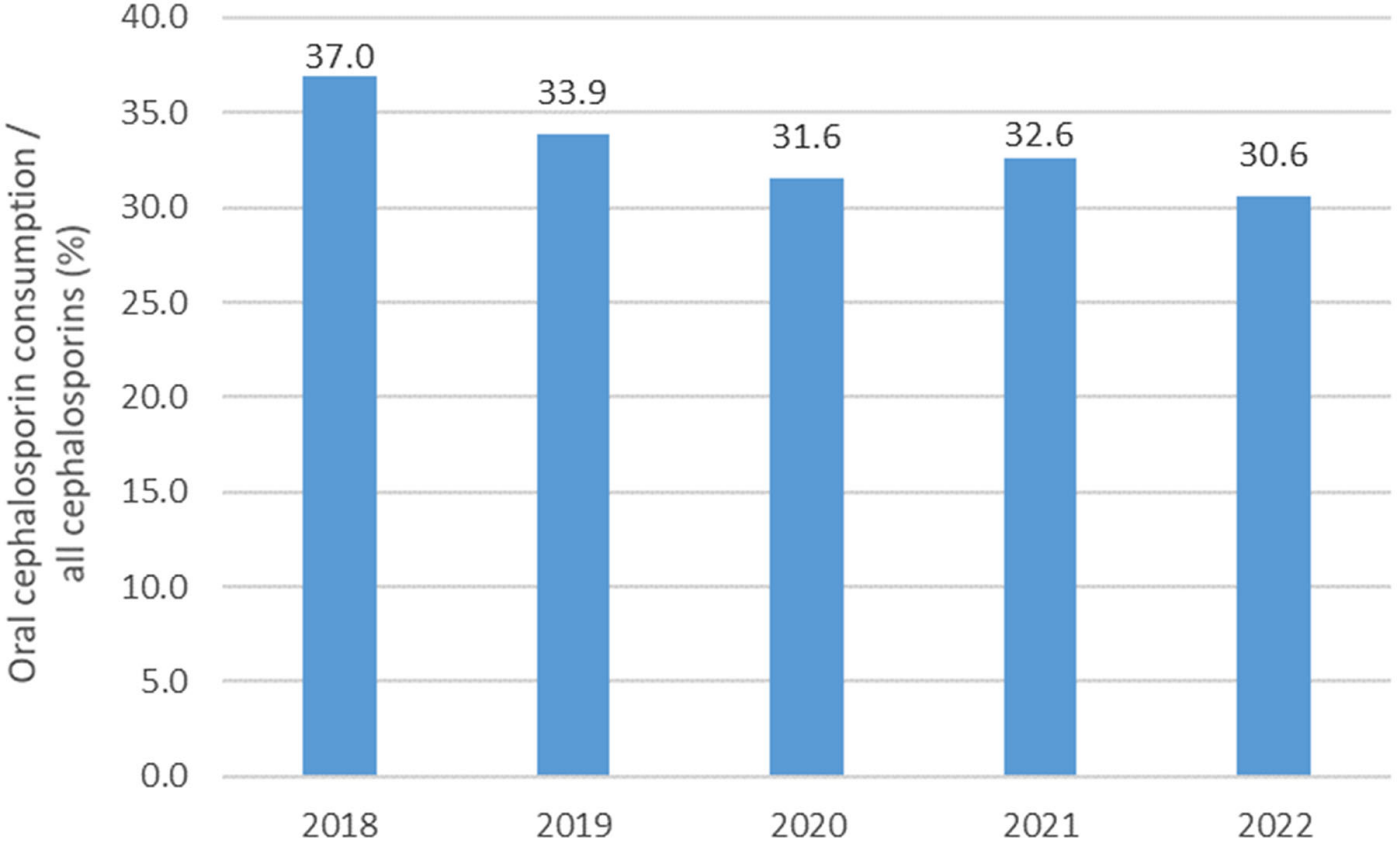
Proportion of oral cephalosporins among all cephalosporins 2018–2022 (N = 220 nursing homes).

The proportion of antibiotics classified in the “critical antibiotics with restricted indications” group^12^ significantly increased (*P* < .01) from 2018 to 2022 (Fig. 2). The proportion of oral cephalosporins consumption among all cephalosporins^13^ significantly decreased during the period (*P* = .02) (Fig. 3).

No trend was seen for the proportion of injectable antibiotics among systemic use antibiotics, which varied between 8.0% and 10.1%, with the highest value in 2020.

## Discussion

This multicentre survey provided useful information on the pattern of antibiotic use in many nursing homes with on-site pharmacies between 2018 and 2022, including the COVID-19 pandemic period. Analysis of trends in antibiotic consumption and in antibiotic indicators is helpful to steer AMS activities.

### Changes in antibiotic consumption in nursing homes over years

The total antibiotic consumption significantly decreased between 2018 and 2022 in the 220 nursing homes included in this study. This trend is consistent with the steady consumption decline reported in nursing homes without on-site pharmacy since 2015 in France.^14^ The 2022 increase may result from the withdrawal of the stringent measures to prevent infections that had been implemented in 2020 leading to a decrease of respiratory infections.^15^ Indeed, antibiotics are often prescribed even for viral respiratory infection,^16^ particularly to elderly people.^17^

During the study period, penicillins represented >60% of nursing-home antibiotic consumption. This proportion is in accordance with a Norwegian study that reported a proportion of 69.6% in 34 nursing homes in 2016.^18^ Penicillins accounted for 30.2% of antibiotic treatments in 2016–2017 during the European prevalence survey in long-term care facilities.^19^ This discrepancy between proportion of DDDs and proportion of treatments is likely linked to the values of DDDs for amoxicillin ± clavulanic acid, which are much lower than the actual prescribed doses in many countries.^20^

The 2 most used antibiotics were amoxicillin-clavulanic acid and amoxicillin. According to the 2018 French national guidelines for treatment of acute infections in elderly in nursing homes, including urinary tract infections (UTIs) and respiratory tract infections,^21^ amoxicillin-clavulanic acid is not a first-line option, except in grade 3 of exacerbation of obstructive lung disease or some acute pneumopathy treatment. However, it remains the most consumed antibiotic. In 2019, it was also the most used antibiotic in a sample of 802 Italian nursing homes, where it accounted for almost 40% of their antibiotic consumption,^22^ compared to 35% in our survey (12.8 of 36.5). In contrast, amoxicillin, which is the most recommended antibiotic depending on the bacteria susceptibility,^21^ was the second most consumed antibiotic. Interestingly, this first-line antibiotic ranked only 11 among most used antibiotics in Italian nursing homes in 2019.^22^

Ceftriaxone was the third most consumed antibiotic and the first among cephalosporins. Indeed, this molecule is recommended in pyelonephritis, prostatitis, bronchitis when the oral route is not possible or in pneumopathy as a second-line treatment.^21^ Cefixime ranked fifth whereas oral third-generation cephalosporins are not recommended for the treatment of acute infections in elderly.^21^

Despite a significant decrease during the study period, pristinamycin was still the fourth most used antibiotic. It is often used as an alternative to amoxicillin or amoxicillin-clavulanic acid in case of allergy in France,^21^ whereas it is hardly used in other countries.^23–25^

The most used fluoroquinolones were ofloxacin followed by ciprofloxacin and levofloxacin (ranking 10 or 11 depending on the year), the latter being the only fluoroquinolone recommended for the treatment of some UTIs in case of penicillin allergy or in prostatitis.^21^ Altogether, fluoroquinolones represented almost 7% of total consumption. This is higher than reported in Norway in 2016 (5.2% of total antibiotic consumption in 34 nursing homes)^18^ but much lower than described in Italy^22^ and the United States.^26^ Although not significantly, fluoroquinolones consumption tended to decrease, in accordance with professional recommendations to restrict the use of these antibiotics. Indeed, fluoroquinolones have a high potential to select resistance, and this is the reason they are classified among the “critical antibiotics with restricted indication” of the 2022 SPILF list^12^ and in the “Watch” list of the 2023 WHO AWaRe index.^27^ Moreover, the European Medicines Agency issued safety alerts in 2018, 2019, and again in 2023 calling to restrict their use, namely in the elderly.^28,29^

### Changes in quality indicators

As information on prescribing appropriateness is not easily available, monitoring antibiotic indicators calculated from quantitative data is promoted by health authorities to evaluate quality of use in nursing homes.^30^ They provide more qualitative information than quantitative data and are less time and resource consuming than practice audits. The consumption of critical antibiotics with restricted indications^12^ has increased, making it very difficult to achieve the expected goal of a 20% reduction by 2025, according to the national action plan to prevent infections and antimicrobial resistance.^6^ As for the proportion of oral cephalosporins among total cephalosporin consumption, despite a significant decrease during the study period, it remains far above the optimal value of 10% and still slightly higher than the “acceptable” 30% threshold in 2022. The third indicator, the proportion of parenteral antibiotics, did not show the decrease expected during the study period; their use must be limited to the situation that required it.^13^ Indeed, using these indicators in addition to the quantitative data on consumption add useful information to steer antimicrobial stewardship activities in nursing homes.

### Changes during COVID-19 pandemic

The decrease in total antibiotic consumption had already started in 2019, but this trend seemed to accelerate during COVID-19 pandemic, from 2020 to 2021. Indeed, the infection prevention and control (IPC) measures implemented during this period limited the number of bacterial infections, especially those transmitted by the respiratory route.^31^ Similar trends in antibiotic use have been reported in 1,944 US nursing homes, in 2020 compared to 2019,^32^ and in Alberta, Canada, but not in Ontario.^33^

Beyond this decrease in total antibiotic consumption, the use of some antibiotics increased during the pandemic in the nursing homes included in our survey. Azithromycin consumption increased sharply as its potential efficacy in COVID-19 treatment was studied at that time.^34^ There was also a slight increase in ceftriaxone use in 2020, probably linked with prescriptions to cover the risk of infection by *S. pneumoniae* and *S. aureus.* ^35^ Indeed, other researchers reported that despite a low proportion (6.9%) of bacterial coinfection in patients hospitalized for COVID-19, 71.9% received antibiotics, often broad-spectrum agents such as third-generation cephalosporins.^36^

These results highlight the need to reinforce antibiotic stewardship actions in nursing homes during pandemic periods to ensure appropriate use of antibiotics. Despite disproval of azithromycin efficacy in the treatment of patients suffering from COVID-19, its consumption remained higher in 2021 and in 2022 than before 2020.

This study had several limitations. We explored antibiotic consumption in nursing homes with on-site pharmacies, which account for 20% of all French nursing homes. These facilities have their own pharmacist and are often hospital-based. For these reasons, they might have easier access to infectious diseases or geriatric experts and might be more likely to benefit from antimicrobial stewardship activities performed by the pharmacist than nursing homes without an on-site pharmacy.^37^ Moreover, as they voluntarily participated in the survey, professionals in the included nursing homes may be more aware of the need for rational antibiotic use. For these reasons, consumption data may not be representative of the entire nursing-home sector in France. Nevertheless, characteristics of antibiotic use described in this survey suggest unwise use, even in nursing homes with on-site pharmacies.

Because prescribing data were not available, no information could be collected on indication or treatment duration. This is why we calculated quality indicators to approximate rational use of antibiotics.

In conclusion, this survey of antibiotic use in nursing homes with on-site pharmacies brought useful information on the pattern of use over years to inform antimicrobial stewardship activities at the national and at the local level. Contrasting results and changes during pandemic periods advocate support for nursing homes professionals to implement antimicrobial stewardship activities tailored to their need and resources.^8^ Implementation of antibiotic multidisciplinary teams is underway in France. They should support professionals in hospitals and in nursing homes to develop actions to improve rational use of antibiotics in close relation with mobile infection control teams. Preventing respiratory tract infections and appropriate treatment of these infections could be a priority. In addition, feedback of surveillance data to nursing home professionals each year may foster awareness and will lead to the implementation of prevention measures and antimicrobial stewardship to increase patient safety.^38,39^ We intend to further promote surveillance of antibiotic use in nursing homes and to help professionals to use their data, including indicators to approach prescribing quality.^6,30^
